# Editorial: Surgical management of spine and spinal cord metastasis

**DOI:** 10.3389/fneur.2025.1639941

**Published:** 2025-07-28

**Authors:** Rouzbeh Motiei-Langroudi, Efstathios Papavassiliou, Andrew Grossbach, Ryan C. Hofler

**Affiliations:** ^1^Department of Neurosurgery, University of Kentucky, Lexington, KY, United States; ^2^Division of Neurosurgery, Beth Israel Deaconess Medical Center, Harvard Medical School, Boston, MA, United States; ^3^Department of Neurosurgery, The Ohio State University Wexner Medical Center, Columbus, OH, United States

**Keywords:** spine metastasis, spinal cord metastasis, radiation, surgery, multidisciplinary treatment

## Introduction

Spine and spinal cord metastases pose significant clinical challenges due to their impact on neurological function, pain, and overall patient quality of life. Treatment paradigms have evolved substantially over recent decades, moving toward integrated, multidisciplinary approaches that combine surgery, radiation therapy, and systemic oncologic treatments. The management of these tumors requires balancing goals of local tumor control, spinal stability, and neurological preservation with the patient's overall prognosis and systemic disease status. Advances in imaging and minimally invasive surgical (MIS) techniques have broadened surgical indications, allowing tailored interventions to decompress neural elements, stabilize the spine, and relieve pain. Radiation therapy remains a cornerstone of treatment in spinal column metastases, particularly with the advent of stereotactic body radiation therapy (SBRT), which delivers high-dose, focused radiation that spares surrounding tissues. SBRT has shown efficacy in local tumor control and is often used either as a primary modality or as an adjunct to surgery (1). However, optimal sequencing and patient selection for surgery vs. radiation remain ongoing challenges that require multidisciplinary input. Despite shifts in surgical indications and techniques over time often shaped by institutional experience and trends, surgery plays a pivotal role in cases with mechanical instability, spinal cord compression or significant neurological compromise, often in combination with radiation (2). In these scenarios, the surgical technique often involves multi-segment fusions (posterior and interbody) added to decompression of neural elements. Moreover, Tan et al. showed an increase in the number of patients managed surgically (added to adjuvant), especially MIS and separation surgery approaches over time, resulting in decreased blood loss, transfusion and improved neurological and functional outcomes. The key in all cases is patients should be managed in a multidisciplinary manner and surgical treatment should be recommended when indicated. Importantly, timely surgical intervention is critical in those with severe spinal cord compression or presence of neurologic deficits, as unnecessary delays in intervention are associated with poorer neurological and functional outcomes. In this regard, Debono et al. compared outcome of elective and urgent surgical intervention for patients with spine metastasis and showed that the 3-month outcomes and overall survival were worse in the emergent group, mostly due to their poorer neurologic baseline. Finally, minimally invasive techniques and novel approaches such as vertebroplasty combined with bone-filling mesh containers (as proposed by Zhan et al.) offer promising strategies to enhance outcomes while minimizing morbidity in those affected by tumor related pain.

Intramedullary spinal cord and conus medullaris metastases represent a particularly rare but complex surgical challenge due to their location and potential for neurological deficits. The goal remains to manage the disease, improve or stabilize neurological function, and palliate symptoms like pain. While gross total resection is desirable, a more realistic (and technically less morbid) subtotal resection followed by radiation is often the paradigm (3). While chemotherapy is often not considered as a first-line treatment for local tumor control in spinal cord metastases, it can be considered in cases refractory to standard therapy. For instance, Wang et al. presented a case of hemangiopericytoma that recurred three times after multiple surgeries and radiation, and chemotherapy and re-radiation could be employed as a successful salvage therapy.

Despite advances in care of these patients, the key in providing the standard of care is pursuing a multidisciplinary approach; as such, Morimoto et al., emphasized the need for active participation of spine surgeons in the care and efficient communication pathways among oncologists, radiation therapists, and surgeons. Byun et al. showed that still several disparities exist in the decision-making process of spine metastasis management among different subspecialties. Therefore, effective management of spine and spinal cord metastases depends on close collaboration among neurosurgeons, orthopedic spine surgeons, radiation oncologists, medical oncologists, internists, rehabilitation specialists and primary care physicians. Divergent perspectives on treatment priorities highlight the necessity of multidisciplinary tumor boards and consensus guidelines to optimize individualized patient care. A coordinated approach ensures comprehensive evaluation of the patient's systemic disease burden, neurological status, and life expectancy, enabling balanced decisions between aggressive local therapy vs. palliative care.

## Conclusion

The treatment of spine and spinal cord metastases are complex. There is an essential need for a patient-centered, multidisciplinary strategy that integrates surgery and radiation (and sometimes chemotherapy) to improve neurological outcomes, maintain spinal stability, and enhance quality of life. As systemic therapies evolve and survival improves, ongoing research and inter-specialty collaboration will be essential to refine treatment algorithms and optimize care for patients with spine and spinal cord metastases.
